# Prevalence and Determinants of Premarital Sexual Practice among Youths in Ethiopia: Based on the Ethiopian Demographic and Health Survey Data

**DOI:** 10.1155/2023/6643797

**Published:** 2023-06-24

**Authors:** Kegnie Shitu, Ayenew Kassie, Maereg Wolde

**Affiliations:** Department of Health Promotion and Health Behaviour, Institute of Public Health, University of Gondar, Gondar, Ethiopia

## Abstract

**Background:**

Premarital sexual practice becomes a common phenomenon among youths in Ethiopia. It is usually associated with unwanted pregnancies, abortions, and sexually transmitted diseases including HIV/AIDS.

**Objective:**

This study is aimed at assessing the magnitude and determinants of premarital sexual practice among Ethiopian youths.

**Methods:**

A community-based cross-sectional study was conducted in all regions of Ethiopia from January 18 to June 27, 2016. A total of 7389 youths with the age range from 19 to 24 were included in the present study. Bivariable and multivariable binary logistic regression analyses were employed to identify factors associated with premarital sex. A 95% CI and *p* value < 0.05 were used to declare statistical significance.

**Result:**

The prevalence of premarital sexual practice was 10.8% (95% CI, 10%–11.5%). Being in the age group of 20-24 (AOR = 3.6, 95% CI (2.8, 4.6)), male sex (AOR = 1.7, 95% CI (1.3, 2.2)), employed (AOR = 1.4, 95% CI (1.03, 1.8)), from pastoral region (AOR = 1.4, 95% CI (1.3,2.4)), having mobile phone (AOR = 1.7, 95% CI, (1.3, 2.3)), ever use of internet (AOR = 1.8, 95% CI (1.3, 2.5)), ever drinking alcohol (AOR = 2.4, 95% CI (1.7, 2.5)), ever chewed khat (AOR = 2.4, 95% CI (1.6, 3.5), and ever tested for HIV (AOR = 1.3, 95% CI (1.1,1.6)) were statistically significant factors associated with premarital sex.

**Conclusion:**

For every 10 youths, at least one of them had sexual intercourse before they got married. Being in the age group of 20-24, male sex, employed, from a pastoral region, having a mobile phone, ever use of the internet, alcohol drinking, khat chewing, and ever tested for HIV were important factors affecting premarital sex. Thus, national sexual education and reproductive health behavior change interventions should give due attention to those groups. Furthermore, adequate education should be given about premarital sexual intercourse when youths come for HIV tests.

## 1. Introduction

In 2019, about 1.2 billion people in the world were between the ages of 15 and 24. More than 200 million live in sub-Saharan Africa, where the largest increase in the proportion of youths is expected in the coming three decades [1]. In Ethiopia, youths aged 15–24 years are more than 15.2 million, contributing to 20.6% of the whole population [2].

This large and productive group of the population is in a state of rapid physical and psychological change with curiosity and the urge to experience new phenomena such as sexual intercourse [3]. In turn, this predisposes them to various sexual risky behaviors such as early sexual initiation, having sex with multiple partners, early marriage, unprotected sex, and premarital sexual practices [4].

Premarital sex is defined as voluntary sexual intercourse between unmarried persons with each other [5]. Globally, youths' sexuality issue has become a recent concern in much research [5]. Recently, the prevalence of premarital sexual practices among youths is increasing in developing countries [6].

Moreover, premarital sexual activity is a debatable issue in its normality. However, in most developing countries like Ethiopia, sexual activities before marriage are mostly done secretly because of fear of social disapproval [7, 8]. On top of that, in such kinds of communities, it is not socially acceptable for youths to buy and use contraceptives before they got married. Due to this, youths are invited to end up with unprotected sex which in turn lets them be suffered from serious health problems such as unintended pregnancies, unsafe abortion, sSexually tTransmitted dDiseases (STDs), hHuman iImmunodeficiency vVirus (HIV), and even death. [9–12].

The prevalence of the premarital sexual practice is highly variable across regions in Ethiopia, which ranges from 22.2% to 71%. [11–17]. Even though there is an increase in youth's sexual activity, preventive reproductive health behaviors, such as condom utilization, are not satisfactory among these groups of the population [12, 14].

As different studies have shown premarital sexual practice is associated with various factors either positively or negatively. These factors include sex [14, 17], older age and peer influence [16], media exposure and work to earn money [17], having a boy/girlfriend [12], residence and family [18], use of social media [14], comprehensive HIV knowledge, alcohol, and smoking [19].

Indeed, there are several studies done in different parts of the country concerning premarital sexual practices among youths. However, none of them used nationally representative data. Therefore, this study is aimed at assessing the prevalence and determinants of premarital sexual behavior of Ethiopian youths based on the nationally representative data of the 2016 Ethiopian Demographic and Health Survey (EDHS). Furthermore, determining the prevalence and identifying factors associated with premarital sexual practice would have great importance for policymakers at the national level to design effective and tailored sexual education programs.

## 2. Methods and Materials

### 2.1. Study Period and Setting

A community-based cross-sectional study was conducted from June 18, 2016, to June 27, 2016. The survey was conducted in all regions of Ethiopia. Ethiopia is the second-most populous country next to Nigeria in Africa with an estimated population of 114 million [20]. About 21% of the population is in the age group of 15-24 [21]. Regarding the health care system, Ethiopia has introduced a health sector transformation plan which is organized by a three-tier health delivery service system led by a ministry, the so-called Ministry of Health of Ethiopia. These metaphorical tires comprise the primary level (health posts, health centers, and primary hospitals), secondary level (general hospitals), and tertiary level (specialized hospitals) [22].

### 2.2. Data Sources, Sampling Techniques, and Population

This study was conducted based on the 2016 Ethiopian Demographic and Health Survey data (EDHS). The survey used the Ethiopian Population and Housing Census, which was conducted in 2007 by the Ethiopia Central Statistical Agency, as a sampling frame. The frame was a complete list of 84,915 enumeration areas (EAs) in which each EAs covers an average of 181 households.

Moreover, EDHS followed a two-stage stratified sampling technique. In the first stage, 645 EAs (202 in urban areas and 443 in rural areas) were selected, whereas, in the second stage, 28 households per cluster were selected from the newly created household listing. All necessary information about the sampling strategy, questionnaire, or other important information is available somewhere else [23] and the pre-print version of this article is vailable at medrivix [24]. Finally, the total weighted samples of 7389 youths were included for this study's analysis.

### 2.3. Study Variables

The outcome variable of this study was premarital sex, which was defined as having sexual intercourse once or more before marriage. To get the outcome variable, we selected never-married young men and women. Then, they were categorized as “Yes” if they had engaged in sexual intercourse and “No” if they had not. The independent variables for the present study were as follows: participants' age, sex, religion, residence, region, education, wealth index, internet use, mobile phone ownership, bank account ownership, substance use (smoking, alcohol drinking, khat, chewing), ever tested for HIV, and media exposure, all of which were extracted from the 2016 EDHS data.

### 2.4. Data Processing and Analysis

Following data extraction, data coding and transformation were done by using Stata version 14.2 software to make the data ready for analysis. Besides, sampling weight was done to adjust for the nonproportional allocation of the sample to strata and regions during the survey process. Since the outcome variable was dichotomy (1 = yes and 0 = no), binary logistic regression was fitted to identify important factors associated with premarital sex. Variables with *p* value < 0.2 in the bivariable logistic regression analysis were entered into multivariable logistic regression analysis.

The Hosmer and Lemeshow test was done to assess the overall model fitness. Furthermore, multicollinearity was tested based on the variance inflation factor (VIF). The VIF of each independent variable was less than 5, which indicated there was no significant multicollinearity between independent variables. Finally, *p* value < 0.05 and 95% confidence intervals were used to determine statistical significance.

## 3. Results

### 3.1. Sociodemographic Characteristics

The median (interquartile range) age of the participants was 18 (16-20) with the age range of 15 to 24. The majority (70%) of them were in the age group of 15-19. More than half (52.6%) of the participants were females. About 4409 (60%) and 749 (10.1%) of the participants had a primary education level and did not attend any formal education at all, respectively (Table 1).

### 3.2. Media Exposure and Behavioral Factors

Among the total participants, only 943 (12.8%) had ever used the Internet, with 358 (4.9%) using the Internet at least once a week. The majority of participants (66.8%) were exposed to at least one of the mass media by which more than half (52%) of them were used to watch television. Approximately 2,733 (37%) and 801 (11%) had ever used alcohol and chewed khat, respectively. Moreover, only 2880 (39%) and 2489 (34%) of the participants knew both methods (condom use and having only one sexual partner only) of HIV prevention and had ever tested for HIV, respectively (Table 2).

### 3.3. Premarital Sex

The overall premarital sex among Ethiopian youths was found to be 10.8% (95% CI (10.1, 11.5)) (Table 3). Of the participants who have a history of sexual intercourse before marriage, nearly half (48.7%) of them started sexual intercourse before they were eighteen.

### 3.4. Factors Associated with Premarital Sexual Practice

Independent variables with a *p* value of less than 0.2 in the univariable logistic regression analysis were entered into multiple logistic regression analyses. These variables were age, sex, education, residence, employment, region, mobile ownership, bank account ownership, wealth, internet, ever alcohol use, ever smoke, ever chew khat, and ever tested for HIV. Moreover, multivariable logistic regression analysis revealed that being in the age group of 15–24, male sex, being employed, being from the pastoral region, having a mobile phone, being an internet user, ever drinking alcohol, ever chewing khat, and ever tested for HIV were positively and significantly associated with premarital sexual practice (Table 4).

## 4. Discussion

The present study is aimed at assessing the prevalence and determinants of premarital sexual practices among youths in Ethiopia. The overall magnitude of premarital sexual practice was 10.8% (95% CI, 10.1–11.5), indicating that for every 10 youths, at least one of them had experienced premarital sex. This study's finding is lower than the findings reported by studies done in Wollega: 28.4% [14]; Tigray: 19% [16]; Debre Markos: 31.3% [12]; and Yabello: 71.9% [11]. The discrepancy may be due to the reason that the present study was based on national data that may reduce the prevalence because of the inclusion of communities with strong social disapproval of premarital sexual practices.

Concerning the factors of premarital sexual practice, being in the age group of 24-29 years, male sex, being employed, living in the pastoral region, having a mobile phone, use of the internet, alcohol drinking, khat chewing, and ever tested for HIV were positively and significantly associated premarital sex.

In this study, participants with the age group of 20-24 years were 5.6 times more likely to be engaged in premarital sex compared to participants with the age group of 15 to 19 years. This finding was similar to what has been reported by a study done somewhere in Ethiopia [16]. This may be explained by the fact that youth with older ages may have independent decision-making ability, and they may have their income to run things by themselves without family recognition and permission.

Being male was another predictor variable that was positively and significantly associated with having premarital sexual activity, where the odds of having premarital sexual intercourse were higher among this group. This finding is in line with findings that have been reported by studies from different parts of Ethiopia [11, 12, 14, 17, 25]. This might be due to that females are expected to keep their virginity until marriage in most parts of the country. On top of that, young males are more free to talk about sexual activities with others than young females [26].

The premarital sexual practice was higher among employed participants by 40% compared to unemployed participants. This is in line with studies from southern Ethiopia [12] and Bahir Dar [17]. This might be because working youth can generate enough money so that they can offer different invitations for the opposite sex and exploit them for sex. In addition, youth who can generate income can have the capacity to pay for sex.

The present study revealed that youths from the pastoral region were 1.8 times more likely to engage in premarital sexual practices than youths from agrarians. This is contradicted by the study from eastern Ethiopia where youths from urban societies were more likely to engage in premarital sexual practice [25]. This may be due to the fact that those people from the pastoral region are mobile and do not have permanent residence. As a result of this, youths from this region may have low awareness of the consequences of premarital sex because of their lower chance to be exposed to health messages due to the mobile nature of their society.

Participants who had either chewed khat or used to drink alcohol were 2.4 times more likely to engage in premarital sexual intercourse than khat and/or alcohol nonusers. This is similar to several studies done previously in the country [8, 10, 12–14]. This might be due to the fact that youths may lose their decision-making ability once they took substances like alcohol and khat, which ultimately lets them engage in premarital sexual practice. Usually, in such risky conditions, sexual practices are done without taking care of it, like without using condoms and having multiple sexual partners, which may then lead them to end up with unwanted/teenage pregnancy, and sexually transmitted infections [8].

Moreover, the probability of having premarital sexual intercourse was 70% and 80% higher among participants who have a mobile phone and are exposed to the internet, respectively. This might be due to the fact that participants having a mobile phone and internet access are at higher risk of being exposed to sexually explicit materials, which in turn may predispose them to have premarital sex [17]. Hence, it is important to make internet services and mobile users to be more selective. Furthermore, awareness of the consequences of inappropriate use of the internet and mobile phone has to be created.

In the present study, the odds of premarital sex were 1.3 times higher among youths who had ever tested for HIV, indicating that those who had tested for HIV were more likely to engage in premarital sexual activity than their counterparts. This is in line with a study done in Wollega, Ethiopia [16]. The possible reason might be due to a common belief that if youths know their HIV status from each other, they are more likely to engage in sexual practices such as unprotected sex.

## 5. Limitations of the Study

One of the limitations of the present study is its cross-sectional nature that precludes us to conclude the causality of the observed associations. In addition to this, recall bias and social desirability bias may affect our estimation since the data were based on self-reports, and the issue is socially sensitive. However, with the aforementioned limitations, the present study tried to picture out the national prevalence of premarital sex and its determinants among youths of the country, which can be consumed by policymakers at the national level concerning youth's reproductive health promotion and disease prevention.

## 6. Strength of the Study

In the presence of the aforesaid limitations, the present study addressed the determinants and prevalence of premarital sex based on nationally representative data so that policymakers or program implementers at the national level can use the evidence generated by the study for the promotion of reproductive health of youths in the country.

## 7. Conclusion

For every 10 youths, at least one of them engaged in premarital sexual intercourse, which is nonnegligible. Being in the age group of 20-24, male sex, being employed, living in the pastoral region, having a mobile phone, ever use of the internet, alcohol drinking, khat chewing, and ever tested for HIV were significant factors associated with premarital sex.

## 8. Recommendation

National sexual education and reproductive health behavior change programs would have paramount importance in reducing the occurrence of premarital sex and, ultimately, its consequences. Moreover, such intervention should give due emphasis to youths who use a substance, have internet access, own a mobile phone, and who are from pastoral regions. Indeed, adequate sexual counseling should be given about premarital sexual intercourse when youths come for HIV tests.

## Figures and Tables

**Table 1:** Sociodemographic characteristics of youths in Ethiopia **(***n* = 7389**).**

Variable	Frequency	Percent (%)
Age		
15-19	5,169	70
19-24	2,220	30
Sex		
Male	3, 500	47.4
Female	3,889	52.6
Religion		
Orthodox	3336	45.1
Muslim	2012	27.2
Protestant	1897	25.7
Other	144	1.9
Residence		
Rural	5482	74.2
Urban	1907	25.8
Region		
Agrarian	6471	87.6
Metropolitans	584	7.9
Pastorals	334	4.5
Education		
No formal education	749	10.1
Primary	4409	59.7
Secondary	1707	23.1
Tertiary	524	7.1
Wealth index		
Poor	2007	27.2
Medium	1324	17.9
Rich	4058	54.9
Owe bank account		
No	6353	86
Yes	1036	14
Owe mobile phone		
Yes	3303	44.7
No	4086	55.3

**Table 2:** Summary statistics of factors of premarital sexual practice among Ethiopian youths (*n* = 7389).

Variable	Frequency	Percent
Internet use		
Never	6446	87.2
Ever	943	12.8
Frequency of Internet use		
Not at all	6446	87.2
Less than once a week	273	3.9
At least once a week	358	4.9
Almost every day	312	4.2
Overall media exposure		
Exposed	4937	66.8
Unexposed	2452	33.2
Specific media exposure		
Ever used to read newspapers/magazines	2180	29.1
Ever used to listen to the radio	3580	48.5
Ever used to watch television	3832	51.9
Ever alcohol use		
No	4656	63
Yes	2733	37
Ever smoke cigarette		
No	7338	99.3
Yes	51	0.7
Ever chewed khat		
No	6588	89
Yes	801	11
Heard about HIV		
Yes	6982	94.5
No	407	5.5
Knowledge of HIV on preventive methods		
Know both methods	2880	39
Do not know	4509	61
Comprehensive HIV knowledge		
No	4163	56.3
Yes	3226	43.7
Ever heard of STI		
Yes	7009	94.8
No	380	5.2
Ever tested for HIV		
No	4900	66.3
Yes	2489	33.7
Awareness of planning methods		
No	254	3.4
Yes	7135	96.6

**Table 3:** Proportion of premarital sex among youths in Ethiopia (*n* = 7389).

Premarital sex	Proportion (%)	95% confidence interval
Yes	10.8	(10.1, 11.5)
No	89.2	(88.5, 89.9)

**Table 4:** Factors associated with premarital sexual practice among Ethiopian youths (*n* = 7389).

Variable	Premarital sex		Bivariable analysis	Multivariable analysis
	Yes (*n* = 795)	No (*n* = 6594)	COR (95% CI)	AOR (95% CI)
	Frequency (%)	Frequency (%)		
Age				
15-19	272 (5.3)	4897 (94.7)	1	1
20-24	523 (23.6)	1697 (76.4)	5.6 (4.7,6.9)^∗∗^	3.6 (2.8,4.6)^∗∗^
Sex				
Male	565 (14.5)	3324 (85.5)	2.4 (1.9, 3)^∗∗^	1.7 (1.3,2.2)^∗∗^
Female	230 (6.6)	3270 (93.4)	1	1
Education				
No education	61 (8.2)	688 (91.8)	1	1
Primary	364 (8.3)	4045 (91.7)	1.01 (.67, 1.5)	1.01 (0.7, 1.7)
Secondary	218 (12.8)	1489 (87.2)	1.6 (1.1, 2.5)^∗^	0.96 (0.6, 1.6)
Tertiary	252 (40.4)	372 (59.6)	4.5 (2.9, 7.3)^∗∗^	1.1 (0.6, 1.9)
Residence				
Urban	312 (16.4)	1595 (83.6)	2 (1.6, 2.5)^∗∗^	1
Rural	483 (8.8)	4999 (91.2)	1	1.1 (0.8, 1.6)
Occupation				
Employed	615 (13.3)	4006 (86.7)	2.2 (1.7, 2.8)^∗∗^	1.4 (1.03, 1.8)^∗^
Unemployed	180 (6.5)	2588 (93.5)	1	1
Region				
Agrarian	634 (9.8)	5838 (90.2)	1	1
Pastorals	35 (10.5)	299 (89.5)	1.1 (.9, 1.3)	1.8 (1.3, 2.4)^∗∗^
Metropolitan	126 (21.6)	458 (78.4)	2.5 (2.1, 3)^∗∗^	1.3 (0.9, 1.7)
Religion				
Orthodox	446 (13.4)	2890 (86.6)	2.2 (.7, 6.7)	1
Protestants	167 (8.8)	1730 (91.2)	1.4 (0.4, 4.3)	1.5 (0.9, 2.3)
Muslims	172 (8.6)	1839 (91.4)	1.3 (0.4, 4.1)	0.98 (0.6, 1.5)
Other	10 (6.7)	135 (93.3)	1	0.64 (0.2, 1.9)
Media exposure				
Exposed	256 (10.4)	2196 (89.6)	1.02 (0.83, 1.3)	
Not exposed	539 (10.9)	4398 (89.1)	1	
Owe mobile phone				
Yes	570 (17.3)	2733 (82.7)	3.6 (2.9, 4.5)^∗∗^	1.7 (1.3, 2.3)^∗∗^
No	225 (5.5)	3862 (94.5)	1	1
Has bank account				
Yes	241 (23.7)	795 (76.3)	3.1 (2.5, 4)^∗∗^	1.1 (0.8, 1.5)
No	554 (8.7)	5799 (91.3)	1	
Wealth index				
Poor	142 (7.1)	1865 (92.9)	1	1
Medium	98 (7.4)	1226 (92.6)	1.1 (.73, 1.5)	0.9 (0.6, 1.4)
Rich	555 (13.7)	3503 (86.3)	2.1 (1.6, 2.7)^∗∗^	1.4 (1, 1.9)
Internet use				
Never used	534 (8.5)	5787 (91.5)	1	1
Ever used	261 (24.4)	807 (75.6)	3.5 (2.8, 4.4)^∗∗^	1.8 (1.3, 2.5)^∗^
Alcohol use				
Yes	457 (16.7)	2277 (83.3)	2.6 (2.1, 3.2)^∗∗^	2.4 (1.7, 2.5)^∗∗^
No	338 (7.3)	4317 (92.7)	1	1
Cigarette smoking				
Never smoke	774 (10.5)	6564 (89.5)	1	1
Ever smoke	21 (41.2)	30 (58.8)	5.7 (2.3, 11.4)^∗∗^	1.6 (0.7, 4)
Khat chewed				
Never chewed	638 (9.7)	5950 (90.3)	1	1
Ever chewed	157 (19.6)	644 (80.4)	2.7 (1.7, 3)^∗∗^	2.4 (1.6, 3.5)^∗∗^
Knowledge of HIV preventive methods				
Know	314 (10.9)	2566 (89.1)	1.03 (0.8, 1.3)	
Do not know	481 (10.7)	4028 (89.3)	1	
Comprehensive HIV knowledge				
Do not have	432 (10.4)	3731 (89.6)	1	
Had	363 (11.3)	2863 (88.7)	1..2 (0.9, 1.4)	
Ever tested for HIV				
No	487 (9.9)	4413 (90.1)	1	1
Yes	308 (12.4)	2181 (87.6)	1.3 (1.03, 1.6)^∗^	1.3 (1.1, 1.6)^∗^

^∗^ *p* value < 0.05 and ^∗∗^*p* value < 0.001.

## Data Availability

The data used for the analysis of the present study is available at https://dhsprogram.com/data/dataset/Ethiopia_Standard-DHS_2016.cfm.

## Abbreviations

AOR: Adjusted odds ratio
AIDS: Acquired immunodeficiency syndrome
CI: Confidence interval
COR: Crude odds ratio
EAs: Enumeration areas
EDHS: Ethiopian Demographic and Health Survey
HIV: Human immunodeficiency virus
STDs: Sexually transmitted diseases.
